# RFC1-related disorders: A case series of 4-aminopyridine and acetyl-DL-leucine treatment

**DOI:** 10.1007/s12311-026-01982-8

**Published:** 2026-04-10

**Authors:** F. Heindl, A. Traschütz, M. Synofzik, T. Wirth, M. Anheim, A. A. Tarnutzer, A. M. Hartmann, D. Rujescu, I. Giegling, K. Feil, C. Adrion, M. Strupp

**Affiliations:** 1 Department of Neurology and German Center for Vertigo and Balance Disorders, Ludwig Maximilians University, Munich, Germany; 2 Division Translational Genomics of Neurodegenerative Diseases, Hertie-Institute for Clinical Brain Research and Center of Neurology, University of Tübingen, Tübingen, Germany; 3 Department of Neurology and Epileptology, Hertie Institute for Clinical Brain Research and Center of Neurology, University of Tübingen, Tübingen, Germany; 4 German Center for Neurodegenerative Diseases (DZNE), Tübingen, Germany; 5 Department of Neurology, The University Hospitals of Strasbourg, Strasbourg, France; 6 University of Strasbourg, Institut de Génétique et de Biologie Moléculaire et Cellulaire (IGBMC), Strasbourg, France; 7 Institut des Neurosciences, University of Strasbourg, Strasbourg, France; 8 Neurology, Cantonal Hospital of Baden, Baden, Switzerland; 9 Faculty of Medicine, University of Zurich, Zurich, Switzerland; 10 Department of Psychiatry and Psychotherapy, Comprehensive Center for Clinical Neurosciences and Mental Health, Medical University of Vienna, Vienna, Austria; 11 University Hospital Ulm (UKU), Ulm, Germany; 12 Institute for Medical Informatics, Biometry and Epidemiology (IBE), Ludwig Maximilians University, Munich, Germany; 13 Department of Neurology, Hospital of the LMU Munich, LMU Munich, Campus Grosshadern, Marchioninistr 15, Munich, 81377 Germany

**Keywords:** *RFC1*-related disorders, CANVAS, 4-aminopyridine, Acetyl-DL-leucine

## Abstract

This observational case series summarizes clinical experiences with 4-aminopyridine (4-AP) and acetyl-DL-leucine (ADLL) in patients with *RFC1*-related disorders treated under named patient use conditions. Clinical data from 30 patients treated at four university centers in Germany, Switzerland, and France were analyzed. Except for three patients previously enrolled in the ALCAT randomized crossover trial, treatments were initiated as part of routine care and were not standardized for dose or duration. Analyses were based on medical records, including patient-reported outcomes and physician assessments. Four patients from Strasbourg underwent SARA and SDFS assessments before and during treatment; SARA total scores for three ALCAT participants were obtained from the original study dataset. Twelve patients received 4-AP only, seven ADLL only, and eleven both treatments at different time points. Median treatment duration was 29 days for 4-AP and 53 days for ADLL. Subjective improvement was reported in 3/22 patients (13.6%) treated with 4-AP and 3/15 (20.0%) treated with ADLL. Objective gait improvement was observed in 2/13 patients (15.4%) on 4-AP and in 2/10 (20.0%) on ADLL. No effects on downbeat nystagmus were observed. Exploratory analyses revealed no significant differences in SARA total scores between ADLL and placebo or baseline; neither SARA nor SDFS scores improved in Strasbourg patients. Responses to 4-AP and ADLL in *RFC1*-related disorders were rare and limited, suggesting that neurodegenerative dysfunction of cerebellar circuits in *RFC1*-disease—in contrast to certain genetic cerebellar disorders such as SCA27B—is unlikely to be improved to clinically relevant thresholds by these agents.

**Supplementary Information:**

The online version contains supplementary material available at 10.1007/s12311-026-01982-8.

## Introduction

*RFC1-*related disorders represent a recently delineated spectrum of neurodegenerative diseases caused by biallelic intronic repeat expansions in the *RFC1* gene [1, 2]. Their clinical phenotype is heterogeneous, ranging from the classical triad of cerebellar ataxia with neuropathy and vestibular areflexia (CANVAS) to more variable phenotypes that overlap with other neurodegenerative conditions, including dysautonomia, parkinsonism, and chorea [3, 4].

To date, pharmacological interventions for *RFC1-*related disorders remain poorly explored. Several agents have been proposed for symptomatic treatment of cerebellar ataxias, including 4-aminopyridine (4-AP) and acetyl-DL-leucine (ADLL) [5]. 4-AP, a voltage-gated potassium channel blocker, was initially introduced in multiple sclerosis, where it improves ocular motor control and motor function, presumably by enhancing conduction in demyelinated nerve fibers [6–8]. Subsequent work has demonstrated clinical efficacy in downbeat nystagmus syndromes and episodic ataxia type 2 (EA2) [9–12], likely through restoration of Purkinje cell excitability [13]; but in particular also in other neurodegenerative genetic conditions with predominant vestibulo-cerebellar dysfunction like spinocerebellar ataxia type 27B (SCA27B; [14–16]). ADLL, an acetylated leucine derivative, has been reported to improve gait variability and overall disease severity, as reflected by reductions in SARA total scores, in case series of patients with various forms of sporadic and hereditary cerebellar ataxias (including SAOA, MSA-C, ADCA, *CACNA1A* mutation, SCA1, and SCA2) treated for at least four weeks and one week, respectively [17, 18]. Conversely, a subsequent investigator-initiated, double-blind, randomized, placebo-controlled crossover trial (ALCAT) did not demonstrate superiority of a six-week ADLL treatment over an equally long placebo treatment [19]. At the cellular level, ADLL has been proposed to exert its effects through stabilization of neuronal membrane potentials [20].

Given the progressive nature of *RFC1-*related disorders and the absence of disease-modifying therapies, systematic evaluation of real-world treatment experiences is warranted. We therefore collected clinical data on named patient treatment with 4-AP and ADLL in a genetically confirmed cohort of patients with *RFC1*-related disorders to provide a foundation for future therapeutic studies.

## Methods

### Case Series Cohort

Patients were recruited from four university centers in Munich: *n* = 19 (P1 – P19), Tübingen: *n* = 5 (P20 – P24), Zurich: *n* = 2 (P25 – P26), and Strasbourg: *n* = 4 (P27 – P30).

Selection criteria for this case series were:

1. genetically confirmed biallelic AAGGG repeat expansions in the *RFC1* gene (identified through either research-based or diagnostic testing), defined according to established diagnostic workflows by failure of flanking PCR amplification together with a positive repeat-primed PCR for the AAGGG motif demonstrating the characteristic saw-tooth pattern [1, 3];
2. a clinical phenotype compatible with *RFC1*-related disease, with evidence of involvement of at least two out of the three core components of the classical CANVAS triad (Supplementary Table 1), irrespective of exact symptom constellation and disease severity;
3. documented treatment with 4-AP, ADLL, or both administered at different time points; and
4. availability of follow-up data on treatment response.

Exclusion was limited to absence of written informed consent and incomplete genotypic or phenotypic data.

Three participants (two from Tübingen and one from Munich) had previously been enrolled in the ALCAT crossover trial [19], and their SARA total scores—encompassing all available assessment time points at baseline, during ADLL treatment, and under placebo—were included in the present analysis.

### Clinical Data Collection and Outcome Assessment

Clinical data from Munich, Tübingen, and Zurich were retrieved retrospectively from medical records, whereas participants from Strasbourg were additionally re-evaluated during the course of this project. Data collection included patient characteristics, results of laboratory investigations (including nerve conduction studies, video head impulse testing, and caloric testing), follow-up information on treatment response, and safety data.

Outcomes were documented using both subjective patient reports and objective assessments of downbeat nystagmus (DBN) and gait function. Subjective improvement was defined as patient-reported benefit documented in the clinical records during routine care; no standardized patient-reported outcome scale was used. Objective evaluations were based on routine neurological examinations performed by trained physicians, and, where available, were supplemented by video-oculography to quantify DBN slow-phase velocity (SPV) as well as by orthoptic assessments. Gait function was assessed during routine neurological examination and included qualitative evaluation of gait stability, need for assistance, and gait velocity. In selected cases, assessment was complemented by a structured evaluation performed by a trained assistant, including observation of gait stability, base of support, and performance under vestibular challenge conditions such as tandem gait, turning, and walking with reduced visual input. Changes under treatment were classified as objective improvement when a physician or trained assistant documented a clear improvement compared with baseline examination, particularly with respect to gait velocity.

Where available, total scores on the Scale for the Assessment and Rating of Ataxia (SARA) and the Spinocerebellar Degeneration Functional Score (SDFS) were obtained under both off- and on-medication conditions. Treatment with the two agents was not standardized with respect to dose or duration and was administered on a named patient use basis (“individueller Heilversuch”) in the absence of regulatory approval in all cases with the exception of three patients previously enrolled in the ALCAT crossover trial.

Given the retrospective real-world design of this case series, no prespecified primary endpoint was defined. The primary descriptive outcome was overall clinical response to treatment, based on patient-reported benefit and physician-assessed changes in gait and downbeat nystagmus. Quantitative measures, including SARA and SDFS scores were analyzed as exploratory outcomes.

### Statistical Analyses

Data were collected using a standardized spreadsheet (Microsoft Excel, version 16.0; Microsoft Corporation, [21]). Descriptive statistical analyses were performed with SPSS Statistics (version 31; IBM Corporation, [22]). Exploratory comparisons of SARA total scores and the generation of patient-specific SARA trajectory plots were conducted using R Statistical Software (version 4.5.1; R Core Team, [23]). For comparisons of SARA total scores across conditions (baseline, placebo, and ADLL), subject-level mean values were calculated by averaging all available measurement time points within each condition. These mean values were then compared using paired t-tests (two-sided). All statistical analyses were exploratory in nature, and a significance level of *p* < 0.05 was applied throughout. For SARA total scores, a minimal clinically important difference (MCID) of 1.5 points was considered, in accordance with recent literature [24].

### Ethics

Following consultation with the Ethics Committee of the Ludwig Maximilians University of Munich, formal ethical approval was not required for this case series, as patients received individualized clinical care and no predefined study protocol was implemented. Responsibility for the justification of named patient use treatment remained with the treating physicians. All patients recruited in Munich provided written informed consent for the use of their genetic and clinical data within the framework of the biobanking project of the German Center for Vertigo and Balance Disorders (project number: 379 − 11). All patients enrolled in Strasbourg provided written informed consent for inclusion in the local prospective registry for sporadic late-onset cerebellar ataxia (ethics committee number: DC-2014-2222). The two patients treated in Zurich had previously been enrolled in a natural history study on *RFC1*-related disease [3] and had therefore already provided written informed consent for study participation. For the three patients who had previously participated in the ALCAT crossover trial, ethical approval and written informed consent had been obtained independently of the present case series [19].

## Results

### Demographics and Clinical Features

This case series comprised 30 patients with *RFC1*-related disorders. Of these, 23 received treatment with 4-AP, 18 were treated with ADLL, and 11 received both agents at different time points. The mean age at onset of first *RFC1*-related symptoms (excluding chronic cough) was 52.8 years (SD 9.6), and the most recent documented clinical evaluation occurred at a mean age of 68.6 years (SD 9.3). The cohort included 14 women (46.7%) and 16 men (53.3%). Baseline functional clinical features are summarized in Table 1.

**Table 1:** Frequency of functional clinical features in 30 RFC1-positive individuals

Functional category	Clinical sign or symptom	*n*/*N*	%	Missing, *n*
*Gait and balance*	Imbalance	30/30	100.0	0
	Gait impairment	30/30	100.0	0
	Worsening without visual control	18/19	94.7	11
	Positive Romberg sign	20/20	100.0	10
	History of falls	20/24	83.3	6
	Oscillopsia	12/16	75.0	14
*Cerebellar features (including imaging)*	Dysarthria	17/25	68.0	5
	Dysdiadochokinesia	15/25	60.0	5
	Upper limb ataxia	24/26	92.3	4
	Lower limb ataxia	20/21	95.2	9
	Fine motor impairment	17/20	85.0	10
	Intention tremor	15/18	83.3	12
	Saccadic pursuit	25/30	83.3	0
	Abnormal saccades	25/30	83.3	0
	Gaze-evoked nystagmus	22/30	73.3	0
	Downbeat nystagmus	22/30	73.3	0
	Impaired VOR suppression	8/24	33.3	6
	Cerebellar atrophy (MRI)	17/27	63.0	3
*Neuropathic features*	Impaired sensation to touch	11/19	57.9	11
	Impaired vibration sense at ankle	26/26	100.0	4
	Abnormal sensory NCS	23/23	100.0	7
*Vestibular features*	Abnormal bedside HIT	26/27	96.3	3
	Abnormal video HIT	13/13	100.0	17
	Abnormal caloric response test	18/18	100.0	12
*Other associated features*	Cognitive impairment	3/21	14.3	9
	Dysautonomia	9/19	47.4	11
	Dysphagia	11/17	64.7	13

Data are presented as number of affected individuals (*n*) over the number of assessed (*N*). Percentages refer to the proportion of affected individuals among those assessed. *N* varies across variables due to missing data. *VOR* vestibulo-ocular reflex, *MRI* magnetic resonance imaging, *NCS* nerve conduction studies, *HIT* head impulse test

### Dosage and Duration of Treatment

Until reassessment, the median duration of treatment was 29 days (interquartile range [IQR] 14–29) in the 4-AP subgroup and 53 days (IQR 12–94) in the ADLL subgroup; treatment durations of the three patients derived from the ALCAT trial were not considered. The most frequently administered daily dose of 4-AP was 20 mg (median 20 mg; range 5−30 mg). ADLL was most commonly administered at a daily dose of 5000 mg (median 5000 mg), with doses ranging from 500 mg to an alternating regimen of 4000–8000 mg per day in a single patient.

### 4-Aminopyridine Response

Patient-reported subjective improvement, assessed dichotomously (yes/no), was observed in 3 of 22 patients (13.6%) receiving 4-AP (P4, P12, and P18); for one of 23 patients of the 4-AP subgroup, subjective response was not documented. Patient P4 (male, 72 years) underwent orthoptic and gait assessments at baseline and 80 min after administration of 5 mg immediate-release 4-AP. A slight, physician-confirmed improvement in gait was observed, whereas DBN was not attenuated and showed a marginal worsening on orthoptic examination. For patient P12 (male, 75 years), an increase in gait velocity was observed at the end of 37 days of treatment with 4-AP; however, no effect on DBN was detected, as determined by orthoptic examination and video-oculography. Patient P18 (female, 61 years) reported the most pronounced subjective benefit, regaining the ability to rise from a chair without assistance at follow-up after six weeks of extended-release 4-AP (20 mg/day). This improvement was not sustained at subsequent reassessments 8 and 11 weeks later, and it remains unclear whether 4-AP was continued beyond the last follow-up. No quantitative assessments were performed in this case.

Among 13 patients who underwent gait assessment, objective improvement was confirmed by the examining physician in two cases (P4 and P12); however, both of which were classified as minor improvements. In a subgroup of nine patients assessed for DBN under both baseline and treatment conditions, no reduction in nystagmus intensity was observed (0/9). Furthermore, four patients recruited at the Strasbourg site underwent systematic evaluation using standardized SARA and SDFS scores during off- and on-treatment periods with 4-AP (20 mg/day; mean treatment duration 35.3 days). None of the patients demonstrated measurable improvement on either scale (Table 2).

**Table 2:** Systematic OFF – ON assessment under 4-AP

ID	Sex (m/f)	Age (years)	4-AP dose (mg/day)	SARA score		SDFS score	
				OFF	ON	OFF	ON
P27	m	74	20	5.0	5.0	3	3
P28	m	71	20	5.5	5.5	3	3
P29	f	67	20	8.0	8.5	4	4
P30	f	52	20	8.5	8.5	3	3

*OFF* off 4-AP, *ON* on 4-AP, *4*-*AP* 4-aminopyridine, *m* male, *f* female, *SARA* Scale for the Assessment and Rating of Ataxia, *SDFS* Spinocerebellar Degeneration Functional Score

### Acetyl-DL-leucine Response>

**Fig. 1:**
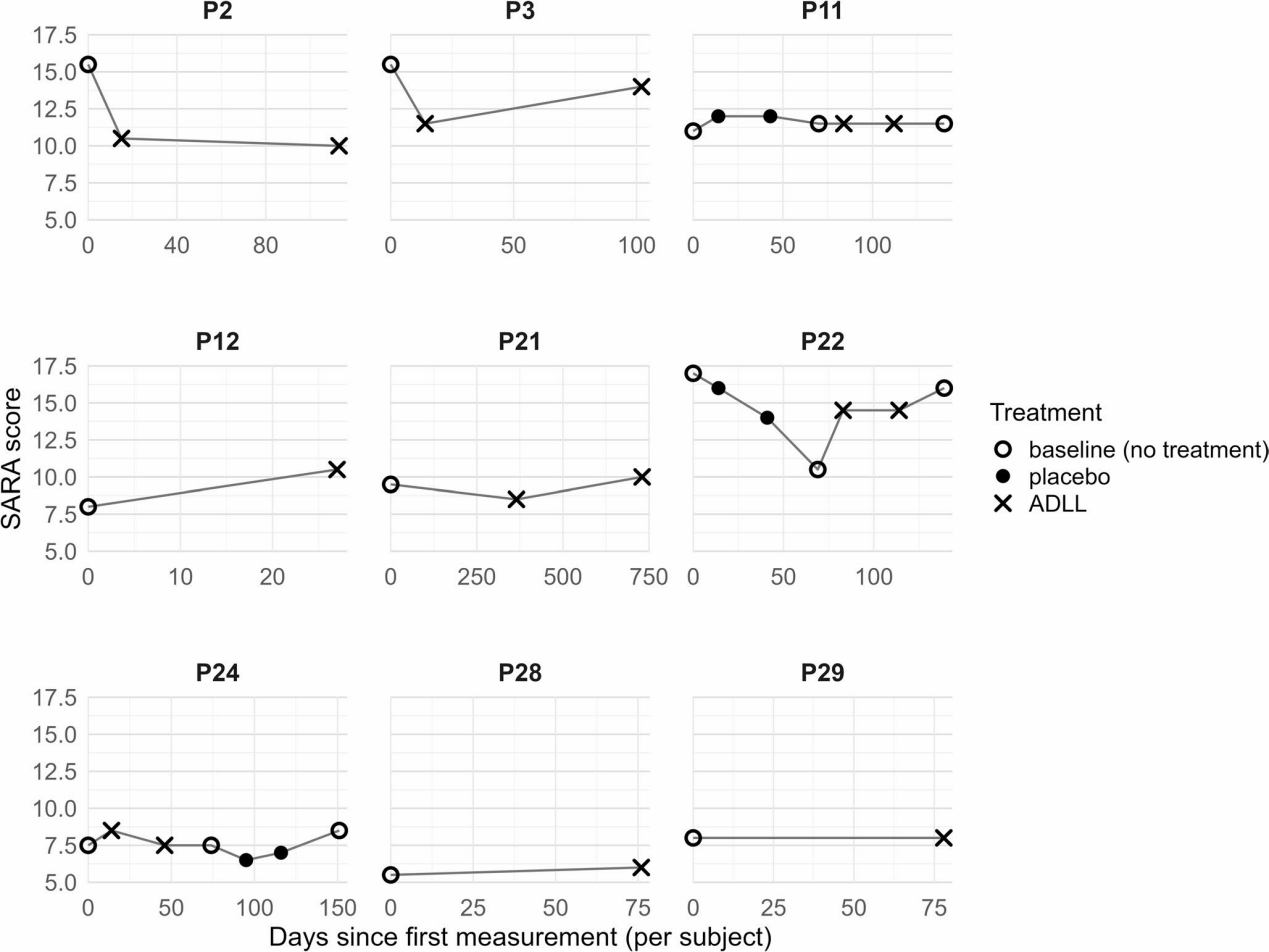
Patient-specific trajectories for the SARA total score under no treatment, placebo, and ADLL. Each panel represents an individual patient treated with ADLL. The y-axis shows the SARA total score, and the x-axis indicates time in days since the first measurement (irregular time points; not standardized). Open circles denote scores obtained under baseline conditions, filled circles denote scores obtained during placebo treatment, and crosses indicate scores obtained during ADLL treatment. SARA total scores from patients P11, P22, and P24 were derived from ALCAT crossover trial

Subjective clinical benefit was reported by 3 of 15 patients (20.0%) receiving ADLL (P3, P16, and P21). Patient P3 (female, 67 years) regained the ability to ambulate without a walker while on ADLL, accompanied by a clinically meaningful reduction in SARA total score of 4 points (from 15.5 to 11.5; Fig. 1). At follow-up approximately three months after initiation of ADLL treatment, the SARA total score had increased to 14.0 points, coinciding with a severe depressive episode that may have confounded the ataxia assessment. DBN was present but not systematically evaluated in this case. In patient P16 (male, 63 years), dysarthria represented the most disabling symptom due to occupational demands. Approximately one year after initiation of ADLL (5000 mg/day), P16 reported marked improvement in speech while receiving ADLL, which led to continued treatment. However, no objective assessments of speech, gait, or nystagmus were available. Patient P21 (male, 73 years) reported improvement in dizziness and cognitive symptoms (“brain fog”) while on ADLL, accompanied by a 1-point SARA reduction (Fig. 1).

Among 10 patients who were systematically evaluated for gait function under baseline and ADLL conditions, physician-confirmed improvement was observed in two cases (P2 and P3; 20.0%) at follow-up after approximately three months of treatment. Attenuation of DBN was not observed in any of the six patients assessed (0/6).

Two patients recruited in Strasbourg underwent off- and on-ADLL assessments using SARA and SDFS scores. In both cases, no reduction in either score was observed (Table 3). To further quantify treatment response to ADLL, subject-level mean SARA scores were calculated for each condition (baseline, placebo, and ADLL) and compared exploratorily. For ADLL vs. placebo (*n* = 3), the mean within-subject difference was 0.08 points (t = 0.14, *p* = 0.90, Cohen’s d_z_ = 0.08). For ADLL vs. baseline (*n* = 9), the mean difference was − 0.55 points (t = − 0.74, *p* = 0.48, Cohen’s d_z_ = − 0.25). Neither comparison reached statistical significance; moreover, the observed differences were below the MCID. Given the small sample size, both analyses were a priori underpowered and yielded wide confidence intervals underscoring the uncertainty of the effect estimates (Table 4).

**Table 3:** Prospective OFF – ON assessment under ADLL

ID	Sex (m/f)	Age (years)	ADLL dose (mg/day)	SARA score		SDFS score	
				OFF	ON	OFF	ON
P28	m	71	5000	5.5	6.0	3.0	3.0
P29	f	67	5000	8.0	8.0	4.0	4.0

*OFF* off ADLL, *ON* on ADLL, *ADLL* acetyl-DL-leucine, *m* male, *f* female, *SARA* Scale for the Assessment and Rating of Ataxia, *SDFS* Spinocerebellar Degeneration Functional Score

**Table 4:** Exploratory paired comparisons of subject-level mean SARA total scores

Comparison	*n* pairs	Mean difference (95% CI)	*p*-value	Cohen’s d_z_
ADLL vs. placebo	3	0.08 (–2.43 to 2.59)	*p* = 0.90	0.08
ADLL vs. baseline	9	–0.55 (–2.24 to 1.15)	*p* = 0.48	–0.25

*SARA* Scale for the Assessment and Rating of Ataxia, *ADLL* acetyl-DL-leucine, *CI* confidence interval

### Safety and Tolerability

Overall, both 4-AP and ADLL were well tolerated. No serious adverse events (SAEs), including seizures, arrhythmias, or hospitalizations attributable to the study medications, were documented. In the 4-AP subgroup, 43.5% (10/23) of the patients reported non-serious side effects, predominantly increased dizziness or vertigo (70%, 7/10); additional complaints included agitation and nausea. In the ADLL subgroup, 16.7% (3/18) of the patients reported mild side effects, including slight subjective memory deficits, foot edema, and increased vertigo. Treatment discontinuation in both groups was primarily driven by perceived lack of benefit and/or sensation of increased dizziness or vertigo.

## Discussion

In this multicenter case series of 30 genetically confirmed *RFC1*-positive patients, we evaluated the real-world effects of 4-AP and ADLL, both administered on a named patient use basis. Only a minority of patients reported subjective clinical benefits, and objective improvements were restricted to gait and observed in a similarly small proportion. The two patients showing minor gait improvement under 4-AP during systematic evaluation (P4 and P12) had also been screened for SCA27B, with negative results in both cases. Notably, neither compound led to attenuation of DBN. Assessments in the Strasbourg cohort likewise demonstrated no measurable change in SARA or SDFS scores under 4-AP (*n* = 4) or ADLL (*n* = 2). In addition, paired comparisons of documented SARA data in the ADLL subgroup revealed no significant differences relative to placebo or baseline. Although these analyses are limited by small sample sizes and must therefore be interpreted with caution, the overall pattern across patient-reported outcomes, physician assessments, and quantitative measures was consistently negative.

The lack of therapeutic effect observed in *RFC1*-positive patients contrasts with prior evidence supporting both agents in other genetically defined ataxias. In episodic ataxia type 2 (EA2), a channelopathy caused by *CACNA1A* mutations [25, 26], 4-AP reduced attack frequency and duration in a placebo-controlled trial [10]. Moreover, 4-AP has been shown to suppress slow-phase velocity in DBN, with the most pronounced effects reported in idiopathic forms [27]. More recently, patients with spinocerebellar ataxia 27B (SCA27B), caused by GAA repeat expansions in *FGF14* and frequently accompanied by DBN [14, 28–30], demonstrated responsiveness to 4-AP, including improvements in patient-reported outcomes, clinician-rated gait and oculomotor signs, and quantitative video-oculography of DBN [14]. In contrast, the absence of any effect on DBN in our cohort suggests that the neurodegenerative dysfunction of (vestibulo-) cerebellar circuits in *RFC1*-related disorders—unlike other genetic vestibulo-cerebellar disorders like SCA27B [14–16]—cannot be improved to clinically relevant thresholds by 4-AP. This likely reflects the view that the neurodegenerative pathology of *RFC1*-related disorders is characterized by more profound Purkinje cell loss and sensory neuronopathy [1, 31], rather than by altered Purkinje cell firing, which is thought to underlie EA2 [26] and, at least as a secondary disturbance, also SCA27B [16, 29, 32].

The experience with ADLL follows a similar pattern. In mixed cohorts of hereditary and degenerative ataxias, the large multicenter, double-blind randomized crossover ALCAT trial demonstrated no superiority of ADLL over placebo on SARA scores [19]. In the post hoc *RFC1* subgroup of the ALCAT trial—including patients P11, P22, and P24 also in our cohort—subject-wise SARA trajectories and the absence of a significant mean difference versus placebo were consistent with the overall trial results. Although recent studies have demonstrated efficacy of N-Acetyl-L-Leucine (NALL) in other neurological conditions, such as Niemann–Pick disease type C [33], which typically present with cerebellar ataxia [34], our findings suggest that not all *RFC1*-patients are likely to derive clinically relevant benefit from ADLL.

The proportion of non-serious adverse events reported under 4-AP treatment in our cohort (43.5%) appears relatively high compared with prior randomized controlled trials of 4-AP in other neurological conditions [10, 27]. However, 70% of reported events reflected increased dizziness, imbalance or vertigo—symptoms intrinsic to *RFC1*-related disease that may be more readily perceived or attributed to treatment. All reported adverse events in our cohort were non-serious and correspond to commonly reported side effects listed in the European Medicines Agency product information [35].

By its nature, this case series has limited inferential power due to heterogeneous dosing regimens and treatment durations, small analytically relevant subgroups, and limited availability of systematic ocular motor and gait assessments, all of which reduce sensitivity to detect subtle treatment effects. Nevertheless, the consistent absence of benefits across patient-reported outcomes, physician assessments, and quantitative measures argues against a meaningful therapeutic effect of either 4-AP or ADLL in *RFC1*-related disease. Given the underlying neurodegenerative pathology, therapeutic approaches aimed primarily at modulating neuronal excitability may be insufficient. Further work should therefore focus on adequately powered randomized trials, early-stage patient cohorts, and sensitive biomarkers to better capture potential treatment effects.

## Supplementary Information

Below is the link to the electronic supplementary material.

Supplementary Material 1 (DOCX 30.5 KB)

## Data Availability

The full datasets generated during the current observational study contain sensitive patient information and are therefore not publicly available. In accordance with local data protection regulations, pseudonymized individual-level data may be made available from the corresponding author upon reasonable request.
