# Supplementary material for: RFC1-related disorders: A case series of 4-aminopyridine and acetyl-DL-leucine treatment
Source: Cerebellum. 2026 Apr 10;25(2):50. doi: 10.1007/s12311-026-01982-8 (PMC13068712; doi:10.1007/s12311-026-01982-8)
Supplement: Supplementary file 1 — Supplementary Material 1 (DOCX 30.5 KB) [file 12311_2026_1982_MOESM1_ESM.docx]

***Supplementary Table 1.*** *Phenotypic core features, available assessments and outcomes at the individual patient level*

| ID | Year | Sex | Center | *RFC1*/ CANVAS phenotype | | | Available assessments (off and on 4-AP and/or ADLL) | | | | | | | 4-AP outcome | | | ADLL outcome | | |
| --- | --- | --- | --- | --- | --- | --- | --- | --- | --- | --- | --- | --- | --- | --- | --- | --- | --- | --- | --- |
|  |  |  |  | CA | N | VA | ALCAT | SARA | SDFS | GA | DBN | ORTH | VOG | subj | gait | dbn | sub | gait | dbn |
| P1 | 1951 | m | M | + | + | + | − | − | − | + | + | − | − | − | − | − | − | u | u |
| P2 | 1944 | f | M | + | + | + | − | + | − | + | − | + | + |  |  |  | u | + | u |
| P3 | 1947 | f | M | + | + | + | − | + | − | + | − | − | − | − | u | u | + | + | u |
| P4 | 1939 | m | M | + | + | + | − | − | − | + | + | + | − | + | + | − |  |  |  |
| P5 | 1956 | f | M | + | u | + | − | − | − | − | + | − | + | − | u | − |  |  |  |
| P6 | 1937 | f | M | + | + | + | − | − | − | + | + | + | + | − | − | − | − | u | u |
| P7 | 1935 | f | M | + | + | + | − | − | − | + | + | + | − | − | u | u | − | − | − |
| P8 | 1938 | m | M | + | + | + | − | − | − | + | − | − | − | − | − | u | − | u | u |
| P9 | 1927 | f | M | + | + | + | − | − | − | − | − | − | − | − | u | u |  |  |  |
| P10 | 1960 | m | M | + | + | + | − | − | − | − | − | − | − | − | u | u |  |  |  |
| P11 | 1951 | m | M | + | + | + | + | + | − | + | − | − | − |  |  |  | − | − | u |
| P12 | 1940 | m | M | + | + | + | − | + | − | + | + | + | + | + | + | − | − | − | − |
| P13 | 1957 | m | M | + | + | + | − | − | − | + | + | + | − | − | u | u | − | − | − |
| P14 | 1951 | m | M | + | + | + | − | − | − | + | + | + | − | − | − | − |  |  |  |
| P15 | 1945 | f | M | + | + | + | − | − | − | − | − | − | − | − | u | u |  |  |  |
| P16 | 1950 | m | M | + | + | + | − | − | − | − | − | − | − |  |  |  | + | u | u |
| P17 | 1947 | m | M | + | + | + | − | − | − | + | + | + | − |  |  |  | − | − | − |
| P18 | 1955 | f | M | + | + | + | − | − | − | − | − | − | − | + | u | u | − | u | u |
| P19 | 1943 | m | M | + | + | + | − | − | − | + | + | + | − | − | − | − |  |  |  |
| P20 | 1965 | f | T | + | + | u | − | − | − | − | − | − | − | − | u | u |  |  |  |
| P21 | 1940 | m | T | + | + | u | − | + | − | − | + | − | − | − | u | u | + | u | − |
| P22 | 1944 | f | T | + | + | u | + | + | − | − | − | − | − |  |  |  | u | u | u |
| P23 | 1953 | f | T | + | + | u | − | − | − | + | + | − | (+)* | u | − | − |  |  |  |
| P24 | 1959 | f | T | + | + | + | + | + | − | − | − | − | − |  |  |  | u | u | u |
| P25 | 1940 | m | Z | + | + | + | − | − | − | + | + | − | − |  |  |  | − | − | − |
| P26 | 1956 | m | Z | + | + | + | − | − | − | + | + | − | − | − | − | − |  |  |  |
| P27 | 1950 | m | S | + | + | + | − | + | + | + | − | − | − | − | − | u |  |  |  |
| P28 | 1953 | m | S | + | + | + | − | + | + | + | − | − | − | − | − | u | − | − | u |
| P29 | 1957 | f | S | + | + | + | − | + | + | + | − | − | − | − | − | u | − | − | u |
| P30 | 1972 | f | S | + | + | + | − | + | + | + | − | − | − | − | − | u |  |  |  |

*The table summarizes the presence of the CANVAS core components (CA, cerebellar ataxia; N, sensory neuropathy; VA bilateral vestibular areflexia), availability of clinical assessments under off- and on-treatment conditions with 4-aminopyridine (4-AP) and/ or acetyl-DL-leucine (ADLL), and documented treatment responses. Assessments include participation in ALCAT crossover trial (ALCAT), gait assessment (GA), downbeat nystagmus evaluation (DBN), orthoptic examination (ORTH), video-oculography (VOG), and ataxia scales (SARA, Scale for the Assessment and Rating of Ataxia; SDFS, Spinocerebellar Degeneration Functional Score). Treatment response is categorized as subjective (subj) or objective improvement in gait (gait) or downbeat nystagmus (dbn). “+” indicates presence or assessment performed; “−” indicates absence or not assessed; “u” indicates unknown, (+)* indicates electronystagmography performed instead of video-oculography. Shaded fields indicate treatment not administered. IDs denote internal case identifiers; participating centers are abbreviated as M (Munich), T (Tübingen), Z (Zurich), and S (Strasbourg); year indicates year of birth.*

Definition of CANVAS core features:

Cerebellar ataxia (CA) was defined by ≥ 2 cerebellar motor signs (dysarthria, limb ataxia [ataxia of upper/ lower limb, intention tremor, dysdiadochokinesia] or fine motor impairment) and/or cerebellar oculomotor dysfunction (≥ 2 of saccadic pursuit, abnormal saccades, gaze-evoked nystagmus, downbeat nystagmus, impaired VOR suppression).

Sensory neuropathy (N) was defined by abnormal nerve conduction studies or, if unavailable, severely impaired vibration sense at the ankles (≤ 3/8 on Rydel-Seiffer bilaterally).

Vestibular areflexia (VA) was defined by bilaterally reduced VOR function (video head impulse test gain < 0.6) and/or bilaterally reduced caloric responses (sum of slow-phase velocities < 6°/s on both sides) and, if both unavailable, bilaterally abnormal bedside head-impulse test.
